# Molecular Cloning and Characterization of a Novel Exo-&beta;-1,3-Galactanase from *Penicillium oxalicum* sp. 68

**DOI:** 10.4014/jmb.2204.04012

**Published:** 2022-07-08

**Authors:** Tong Zhou, Yanbo Hu, Xuecui Yan, Jing Cui, Yibing Wang, Feng Luo, Ye Yuan, Zhenxiang Yu, Yifa Zhou

**Affiliations:** 1 Department of Endocrinology and Metabolism, Department of Respiratory Medicine, The First Hospital of Jilin University, Changchun 130021, P.R. China; 2 School of Food Sciences and Engineering, Chang Chun University, Changchun 130022, P.R. China; 3 Engineering Research Center of Glycoconjugates, Ministry of Education, Jilin Provincial Key Laboratory of Chemistry and Biology of Changbai Mountain Natural Drugs, School of Life Sciences, Northeast Normal University, Changchun, P.R. China; 4 Central Laboratory, Changchun Normal University, Changchun Jilin province, P.R. China

**Keywords:** Exo-β-1, 3-galactanase, glycoside hydrolase family 43, *Penicillium oxalicum*, larch wood arabinogalactans

## Abstract

Arabinogalactans have diverse biological properties and can be used as pharmaceutical agents. Most arabinogalactans are composed of β-(1→3)-galactan, so it is particularly important to identify β-1,3-galactanases that can selectively degrade them. In this study, a novel exo-β-1,3-galactanase, named PoGal3, was screened from *Penicillium oxalicum* sp. 68, and hetero-expressed in *P. pastoris* GS115 as a soluble protein. PoGal3 belongs to glycoside hydrolase family 43 (GH43) and has a 1,356-bp gene length that encodes 451 amino acids residues. To study the enzymatic properties and substrate selectivity of PoGal3, β-1,3-galactan (AG-P-I) from larch wood arabinogalactan (LWAG) was prepared and characterized by HPLC and NMR. Using AG-P-I as substrate, purified PoGal3 exhibited an optimal pH of 5.0 and temperature of 40°C. We also discovered that Zn^2+^ had the strongest promoting effect on enzyme activity, increasing it by 28.6%. Substrate specificity suggests that PoGal3 functions as an exo-β-1,3-galactanase, with its greatest catalytic activity observed on AG-P-I. Hydrolytic products of AG-P-I are mainly composed of galactose and β-1,6-galactobiose. In addition, PoGal3 can catalyze hydrolysis of LWAG to produce galacto-oligomers. PoGal3 is the first enzyme identified as an exo-β-1,3-galactanase that can be used in building glycan blocks of crucial glycoconjugates to assess their biological functions.

## Introduction

Arabinogalactans (AGs) are a particularly interesting class of polysaccharides found in a range of plants. AGs are usually divided into two structural types: AG-I and AG-II. In terms of their backbone structures, AG-I is predominantly β-D-(1→4)-galactan, whereas AG-II is β-D-(1→3) and/or (1→6)-galactan. Large quantities of AGs are present in Larix trees [1-3], and have been shown to possess diverse biological properties, including immunological activity [4], antitumor [5], and antiviral effects [6]. AGs from *Larix laricina* have been reported to play a unique role in reducing the incidence of the common cold [7]. In addition, AGs have been approved by the US Food and Drug Administration (FDA) for use as dietary fiber. AG-mediated biological activity is generally associated with its monosaccharide composition, type of glycosidic linkage, molecular weight, as well as the number and type of substituents and branched chains. Therefore, analysis of the complex fine structure and function is a significant problem in glycobiology.

Glycoside hydrolase is an important enzyme that is widely used in the study of polysaccharides [8]. Up to now, about 171 glycoside hydrolase families have been reported in the CAZy database [9]. β-Galactanases are a broad group of enzymes that hydrolyze glycosidic bonds in plant-derived galactans, and have been widely used in the analysis of polysaccharide structures, construction of medicinal plant fingerprints, preparation of galactooligosaccharides, and food quality improvement [10-12]. This group of enzymes can be divided into β-1,3-galactanase, β-1,4-galactanase and β-1,6-galactanase according to the structure of their substrates [13]. At present, there are many studies on the preparation and functional analysis of β-1,4-galactanase, whereas investigations with β-1,3- and β-1,6-galactanases are relatively rare [14, 15]. Therefore, it has become crucial to the field to prepare and characterize β-1,3- and β-1,6-galactanases.

β-1,3-Galactanase primarily functions as an endo- and exo-β-1,3-galactanase, with the latter being a key enzyme in the analysis and degradation of AGs. This enzyme specifically cleaves β-(1→3)-galactosidic linkages in the β-3,6-galactan core of AG-II [16]. Presently, there are eight exo-β-1,3-galactanases in the NCBI and CAZy databases. The group consists of Il3Gal from *Irpex lacteus* (GenBank: BAH29957.1) [17, 18], BLLJ_1840 from *Bifidobacterium longum* subsp. *longum* JCM1217 (GenBank: BAJ67504.1) [19], Fo1,3Gal from *Fusarium oxysporum* (GenBank: BAG80558.1) [20], Pc1,3Gal43A from *Phanerochaete chrysosporium* (GenBank: BAD98241.1) [21, 22], Ct1,3Gal43A from *Hungateiclostridium thermocellum* ATCC27405 (GenBank: ABN51896.1) [23, 24], Sa1,3Gal43A from *Streptomyces avermitilis* MA-4680=NBRC14893 (GenBank: BAC69820.1) [25] and SGalase2/SGalase1 from *Streptomyces* sp. 19 (GenBank: AFH55135.1 / AFH55134.1) [26]. All of them have been cloned and heterologously expressed in *E. coli* and *P. pastoris* GS115, and their enzymatic properties and potential applications to arabinogalactan structures have been explored. However, a limited number of exo-β-1,3-galactanases have been characterized, primarily due to their low expression levels, problems with viability, and resistance to acids and bases. Thus, it is essential to obtain a variety of exo-β-1,3-galactanases to prepare oligosaccharide fragments and analyze the structures of AGs. In this study, we biochemically characterized a novel exo-β-1,3-galactanase (PoGal3) from *Penicillium oxalicum* sp. 68, that can specially hydrolyze larch wood arabinogalactans (LWAG), leading to production of galactose and β-1,6-galactooligomers. Therefore, this enzyme is a potentially effective tool for use in the structural analysis of these polysaccharides.

## Materials and Methods

### Strains and Reagents

Strain *P. oxalicum* sp. 68 was isolated from soil in Changbai Mountain (Jilin Province, China), and stored in China General Microbiological Culture Collection Center (collection number CGMCC 7.328) [27]. *P. pastoris* GS115 and *p*PICZα A (Novagen, USA) were used as host and expression vectors, respectively. LWAG, debranched arabinan, arabinan (sugar beet), wheat arabinoxylan, galactomannan, and galactan (potato) were from Megazyme International Ireland Ltd. (Ireland). DNA purification and plasmid isolation kits were from Tiangen Biotech (China). All other chemicals and reagents were of analytical grade.

### Construction of Plasmids and Strains

Total RNA was extracted from *P. oxalicum* sp. 68, and the mycelia were frozen in liquid nitrogen, homogenized with mortar and pestle, and extracted with Trizol. Single-strand cDNA was synthesized from 2 μg of total RNA using a reverse transcriptase (Cat. No. M531A, Promega, USA), and oligo (dT)-adaptor primer (Takara, Japan). Two primers, *pogal3*-F (5-CCGCTCGAGATGTATCTTGGAAGAGGCTTC-3) and *pogal3*-R (5-TCCCCGCGG TTACTACTGCTGCGGTACTAA-3), were designed based on the amino acid sequence of exo-β-1,3-galactanase, which was predicted in a previous paper [28]. PCR was performed using DreamTaq Green PCR Master Mix (Thermo Scientific, USA), and the program was as follows: 98°C for 30 s, 30 cycles of 98°C for 10 s, 68°C for 45 s, 72°C for 1 min 30 s, and final extension at 72°C for 10 min. The PCR product and *p*PICZα A were digested with SacII and XhoI, and the gene was ligated with *p*PICZα A to generate the recombinant plasmid *p*PICZα A-*pogal3*. All enzymes used were from New England Biolabs (USA). Restriction enzyme digestions, ligations and transformations were performed according to the suppliers’ recommendations. Electroporation and selection of transformants were carried out by using MD and G418. The selected clone was cultured in BGMY medium at 30°C for 4 days, with methanol being supplemented (0.5%) every 24 h during the induction period. Cells were harvested by centrifugation at 8000 *×*g for 10 min, and the crude enzyme was found to be present in the supernatant.

### Expression and Purification of Recombinant PoGal3

The culture medium (200 ml) was centrifuged at 8,000 *×*g for 10 min, and the supernatant was collected and precipitated with 80% ammonium sulfate, dissolved and dialyzed against 50 mM Na-acetate buffer (pH 4.5). The protein was passed through a 1.6 × 100 cm Sephacryl S-100 HR column (GE Healthcare, USA). Adsorbed proteins were then eluted by 50 mM Na-acetate buffer at a low rate of 0.15 ml/min. Purified PoGal3 was analyzed by sodium dodecyl sulfate-polyacrylamide gel electrophoresis (SDS-PAGE) on 10% separating gel [29], and protein concentrations were determined by using the method of Bradford with bovine serum albumin (BSA) as the standard.

### Characterization of Recombinant PoGal3

The pH dependence of purified PoGal3 was determined in different buffers (50 mM final concentration) at different pH values (pH 2.0-6.0, Na-acetate buffer; pH 6.0-8.0, Na_2_HPO_4_-NaH_2_PO_4_ buffer; pH 8.0-11.0, Glycine-NaOH buffer) using AG-P-I as substrate. The pH stability was investigated under standard assay conditions following incubation of the purified enzyme for 24 h at 4°C in the buffer without substrate.

The optimum temperature was determined by measuring enzymatic activity at pH 5.0 over the temperature range of 20-70°C. Temperature stability was measured by analyzing residual activity after incubation of aliquots of the enzyme at different times and different temperatures.

The effects of metals on PoGal3 activity were tested in the presence of 50 mM metal ions for 12 h at 40°C. The remaining activity was determined using AG-P-I as substrate, as described before, and activities are expressed as a percentage of activity obtained in the absence of the compound.

### Substrate Specificity Analysis of PoGal3

Substrate specificity was determined using various galactans as substrates. Hydrolytic activity was determined at 40°C in Na-acetate buffer, pH 5.0, with 0.5% (w/v) polysaccharides as substrates and 5 μg PoGal3. After incubation for the desired reaction time, liberated reducing sugars were measured by the method of Somogyi [30]. To determine hydrolysates of different galactose-containing polysaccharides, the reaction mixture containing 50 μl of a 4 mg/ml substrate solution, 140 μl of 20 mM Na-acetate buffer (pH 5.0), and 10 μl of PoGal3 (100 μg/ml) was incubated for 12 h at 40°C, and enzymatic productions were analyzed by using high-performance anion-exchange chromatography (HPAEC).

### Preparation of AG-P-I

LWAG (4 mg/ml) was dissolved in 100 ml distilled water containing 100 mm sodium periodate solution in the reaction system. The sample was then protected from light at 4°C. The OD_223nm_ was maintained and 4 ml ethylene glycol was added to the solution to terminate the reaction [31]. The reaction system was repeatedly dialyzed with a dialysis bag having a pore diameter of 3 KDa and freeze-dried. AG-P-I (1 mg) was analyzed by PMP pre-column derivatization and HPLC detection [32].

### Methylation Analysis

The methylated sample was analyzed by GC-MS with a Technologies 7890B GC and 5977B MSD equipped with a DB-1 capillary column (0.25 mm × 30 m). Conditions of the GC column were as follows: initial temperature of 120°C for 1 min, then 3°C/min to 210°C for 2 min, and then 10°C/min to 260°C for 4 min; the injection temperature was 250°C. Nitrogen was used as the carrier gas and maintained at 1.2 ml/min. The percentage of the methylated sugars was calculated as ratios of the peak areas.

### ^13^C NMR Spectra

AG-P-I (20 mg) was dissolved in D_2_O (0.5 ml), and natural abundance ^13^C NMR spectra were obtained using a Bruker Avance 600 MHz spectrometer (Bruker Inc., Germany) operating at 150 MHz for carbon. Chemical shifts were given in ppm with acetone as the internal chemical shift reference.

### Nucleotide Sequence Accession Number

Sequences for the 18S rRNA genes from *P. oxalicum* sp. 68 were deposited in GenBank under accession number KR349463.

## Results

### Cloning, Expression and Purification of Recombinant PoGal3

Exo-β-1,3-galactanase from glycoside hydrolase family 43 (GH43), has proved to be a key enzyme for the degradation of pectin. *P. oxalicum* has been reported to produce pectin-degradation enzymes in culture media when using pectin as the sole carbon source [33]. In this study, the GH43 gene *pogal3* from *P. oxalicum* was cloned into expression vector *p*PICZα A (XhoI/SacII), and *P. pastoris* GS115 was chosen as the host cell to produce recombinant PoGal3. Gene *pogal3* consists of 1,356 bp (451 amino acid residues) with a theoretical molecular mass of 48.5 kDa and pI value of 6.6 (http://web.expasy.org/computepi/).

**Fig. 1:**
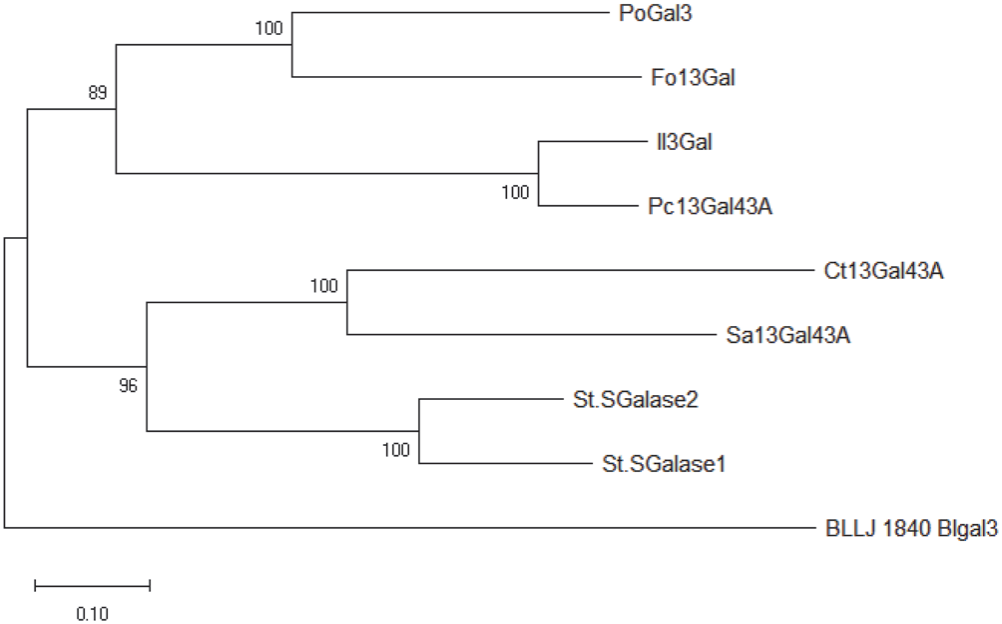
Phylogenetic analysis of amino acid sequences of PoGal3 and eight exo-β-1,3-galactanases from different sources. exo-β-1,3-galactanase Il3Gal (GenBank: BAH29957.1), BLLJ_1840 (GenBank: BAJ67504.1), Fo1,3Gal (GenBank: BAG80558.1), Pc1,3Gal43A (GenBank: BAD98241.1), Ct1,3Gal43A (GenBank: ABN51896.1), Sa1,3Gal43A (GenBank: BAC69820.1) and SGalase2/SGalase1 (GenBank: AFH55135.1 / AFH55134.1).

Analysis of PoGal3 with BlastP and Pfam identified the protein as a member of the GH43 family, which has a GH43-6 functional domain and a CBM functional domain (CAZy database: http://www.cazy.org/CAZY), Residues 1 to 20 are predicted to be a signal sequence. PoGal3 was the first enzyme shown to have activity as an exo-β-1,3-galactanase in *P. oxalicum*. Previously, eight exo-β-1,3-galactanases from different sources had been characterized. The amino acid sequence of PoGal3, as well as those of previously published exo-β-1,3-galactanases, were analyzed. As shown in Fig. 1 and Fig. S1, amino acid sequence alignments and phylogenetic analyses indicate that PoGal3 is highly similar to Fo1,3Gal (GenBank: BAG80558.1) and Pc1,3Gal43A (GenBank: BAD98241.1), with less similarity to Sa1,3Gal43A (GenBank: BAC69820.1), Ct1,3Gal43A (GenBank: ABN51896.1) and BLLJ_1840 (GenBank: BAJ67504.1).

**Fig. 2:**
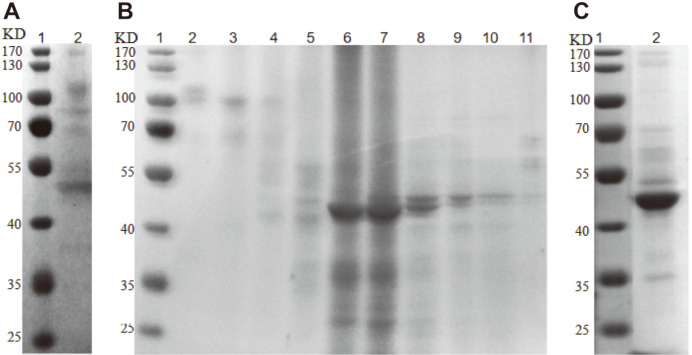
SDS-PAGE analysis of recombinant PoGal3. (**A**) Expression of recombinant PoGal3 in GS115 after ammonium sulfate precipitation method: lane 1, protein markers; lane 2, PoGal3 after ammonium sulfate precipitation method. (**B**) Purification of recombinant PoGal3 by Sephacryl HR S-100: lane 1, protein marker; lane 2 to lane 8, elated by Na-acetate buffer (50 mM). (**C**) Purification of recombinant PoGal3 after two-step chromatography: lane 1, protein markers; lane 2, PoGal3 after two-step chromatography.

To systematically study the activity of recombinant PoGal3, the enzyme was purified by ammonium sulfate precipitation and Sephacryl S-100 HR. As shown in Fig. 2, the expressed protein shows essentially a single band after a two-step chromatography protocol, corresponding to an apparent molecular mass of ~ 48.5 kDa.

### Preparation of AG-I-P

**Fig. 3:**
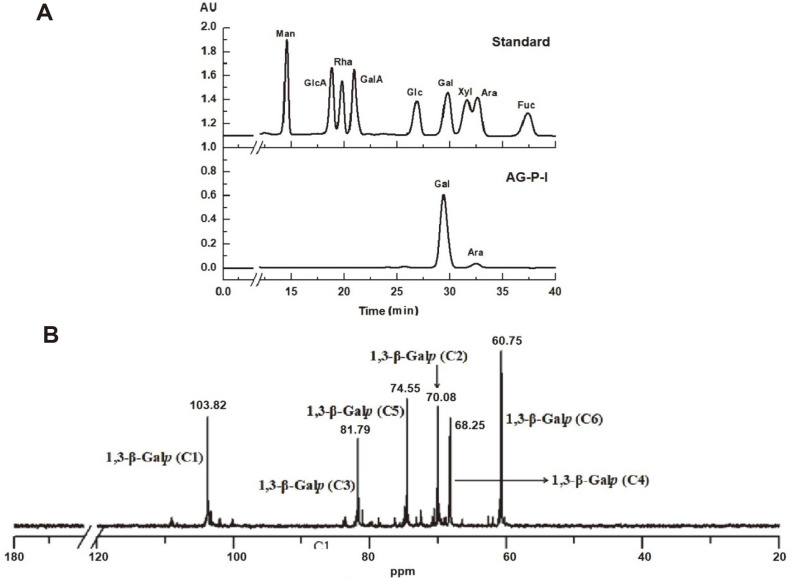
Structural characterization of AG-P-I by HPLC (**A**) and ^13^C NMR (**B**).

To assess the enzymatic properties and substrate selectivity of PoGal3, β-1,3-galactan (AG-P-I) from LWAG was prepared by periodate oxidation and Smith degradation. After dialysis and lyophilization, purified AG-P-I was obtained with 28.25% yield. HPLC results showed that AG-P-I is primarily composed of galactose with a small amount of arabinose (Fig. 3A). The structure of AG-P-I was analyzed by ^13^C NMR and GC-MS, as depicted in Fig. 3B and Table 1. Methylation analysis indicated that the Gal residues are mostly present as terminal and 1,3-linked units, in addition to containing a small amount of 1,6-linked Gal and 1,3-linked Ara residues (Table 1). The content of 1,3-linked Galp was ~58.5%, with 20.2% being found at terminal positions. The chemical shifts of major resonances were assigned based on literature values [34, 35]. The anomeric carbon signals of β-1,3-linked Galp were identified at 103.82 ppm, and the C-2, C-3, C-4, C-6 of 1,3-linked Galp were found to resonate at 70.08 ppm, 81.79 ppm, 68.25 ppm and 60.75 ppm, respectively. Overall, these results are consistent with AG-P-I being composed of β-1,3-galactan, making it suitable as a substrate for the study of PoGal3.

### Biochemical Characterization of Recombinant PoGal3

**Fig. 4:**
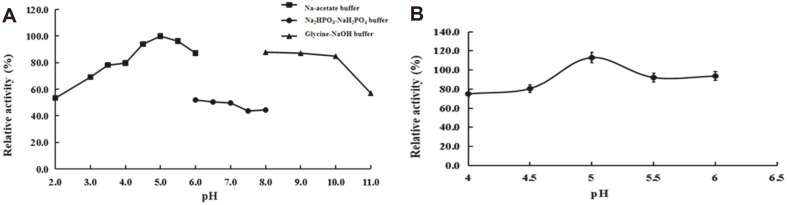
The effect of pH on activity (**A**) and stability (**B**) of PoGal3 used AG-P-1 as substrate. The activity of the enzyme before incubation was defined as 100%. Results are presented as means±standard deviations (*n* = 3).

**Fig. 5:**
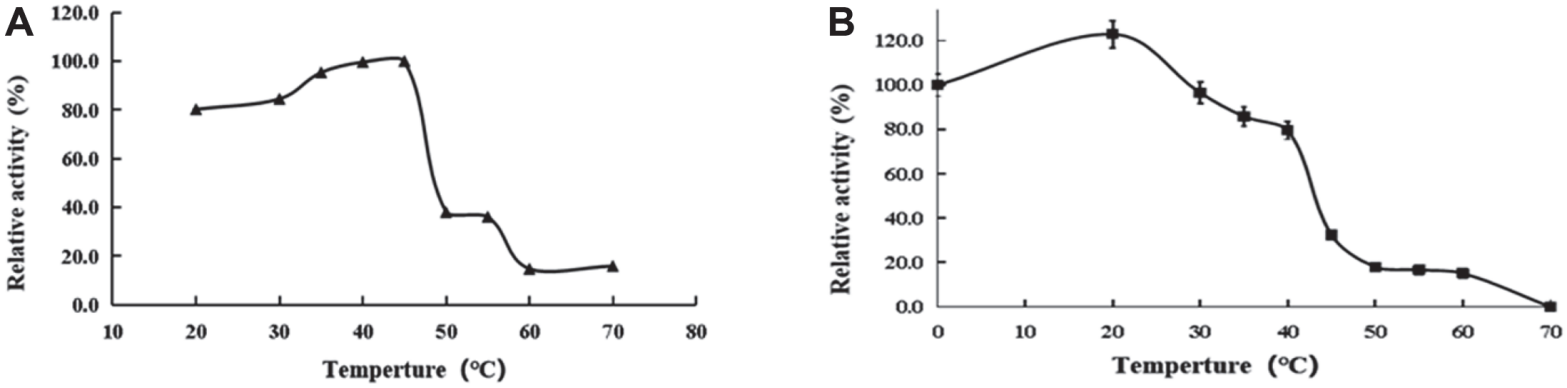
The effect of temperature on activity (**A**) and stability (**B**) of PoGal3 using AG-P-1 as substrate. The activity of the enzyme before incubation was set as 100%. Results are presented as the mean ± standard deviation (*n* = 3).

Using AG-P-I as substrate, we examined the activity of PoGal3 over a pH range from 2.0 to 11.0. Even though our results showed that the optimal pH was 5.0, the enzyme was stable from pH 4.5 to 6.0 and retained > 80% of the initial activity up to 12 h (Fig. 4). The effect of temperature on the enzyme activity was investigated at optimal pH, and the maximum activity was observed at 40°C. The thermostability of PoGal3 was investigated from 20 to 70°C, and as shown in Fig. 5, PoGal3 is stable up to 40°C, with approximate 80% of its activity remaining when held at 40°C for 2 h. Nevertheless, the activity significantly decreased above 40°C. Therefore, for our biotransformation and structural analyses, we used the reaction conditions of pH 5.0 and 40°C.

Certain inorganic and organic factors greatly affected PoGal3 activity and the effects of metal ions on PoGal3 activity are shown in (Table 2). Zn^2+^ had the strongest promoting effect on activity, increasing it by 28.6%, whereas DTT, K^+^, Na^+^, Ba^2+^ and Ca^2+^ did not affect the activity. In contrast, Hg^2+^ and Cu^2+^ significantly inhibited enzymatic activity. While there are few reports about the effects of metal ions on β-1,3-galactanase activity, the diverse effects of metal ions on the enzyme activity may be attributed to interactions with amino acid residues of the enzyme that correlate with protein structural alterations, which could produce either positive or negative effects on the catalytic rate or other enzymatic properties.

### Substrate Specificity of Recombinant PoGal3

**Fig. 6:**
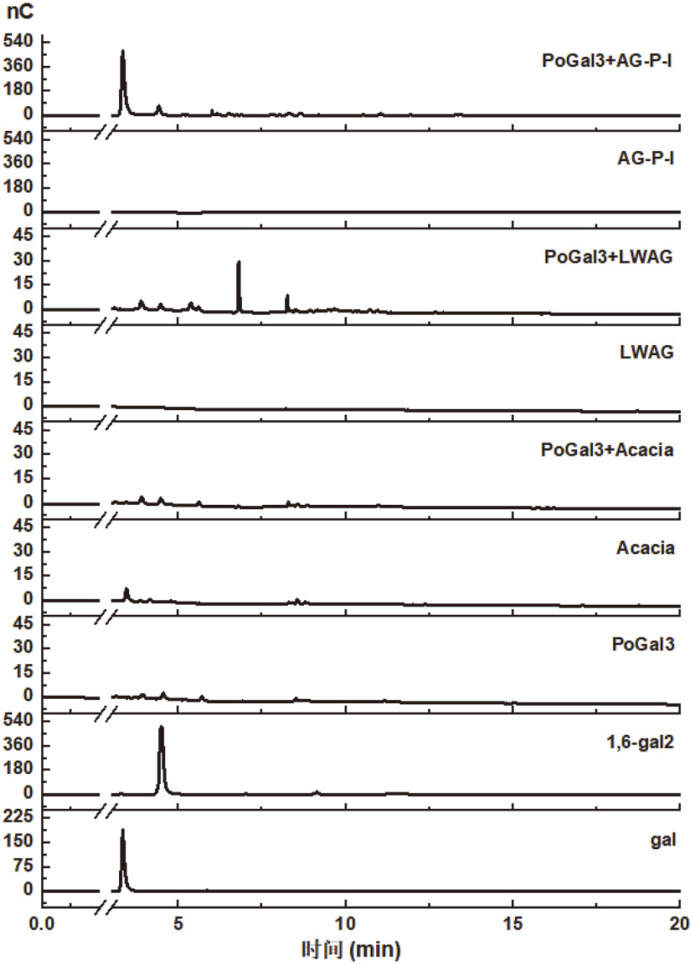
HPAEC of degradation products of different polysaccharides hydrolyzed by PoGal3. Reactions were incubated with recombinant enzyme at 40°C for 12 h. The bottom of each graph was the standard sample of galactose or 1,6- galactoise, and oligosaccharides produced in the reaction were detected by HPAEC at 0 h and 12 h.

The specificity of PoGal3 was assayed using different types of polysaccharides. As shown in Table 3, PoGal3 acts on AG-P-I to release reducing sugar, and has little activity towards β-1,3-glucan, carboxymethyl cellulose (CMC), LWAG, and laminarin. While PoGal3 had no activity towards most polysaccharides, such as β-1,4-galactan, arabinan, oat xylan, etc., PoGal3 does function as an exo-1,3-galactanase, with the highest catalytic activity being observed on AG-P-I. To understand PoGal3’s mechanism of action, we employed three AGs with different molecular weights, including *Acacia* (425 kDa), LWAG (27 kDa) and AG-P-I (6.5 kDa). Results with HPAEC (Fig. 6) show that PoGal3 degrades LWAG to produce oligosacchrides, whereas it acts on AG-P-I to produce galactose and β-1,6-galactobiose. Our results also indicate that PoGal3 catalyzes the hydrolysis of β-1,6-galactosyl side chains in LWAG, producing galactooligomers. Based on our structure information of AG-P-I shown in Fig. 3, we can infer that PoGal3 acts on AG-P-I in an exo-fashion, because the enzymatic products of AG-P-I are mainly galactose with little β-1,6-galactobiose. This is consistent with AG-P-I being mostly composed of β-1,3-galactan with little β-1,6-linked Gal*p* residue. Exo-β-(1→3)-galactanase activity was first observed around 1990 [17], after which there have been few reports on exo-β-(1→3)-galactanase. In this regard, it was first rigorously characterized by Ichinose and co-workers in 2005 [21]. Our results suggest that PoGal3 exhibits unique enzymatic properties on AGs with low molecular weight, which may be potentially useful as a tool to prepare oligosaccharide and analyze the structure of arabinogalactans.

## Discussion

In the present study we have reported on the recombinant enzyme PoGal3 from the GH43 family in *P. oxalicum* sp. 68. PoGal3 hydrolyzes galactose-containing polysaccharides and oligosaccharides. To produce PoGal3 efficiently, *P. pastoris* GS115 was selected as the expression host instead of E.coli BL 21 (DE3), primarily because the yeast expression system was thought to be more suitable for heterologous expression of genes from fungi that can perform glycosylation, for example. This system was found to be conducive for the expression of genes from fungi into soluble proteins, such as *P. oxalicum*, *Flammulina velutipes* and *Aspergillus aculeatus* [27, 36, 37]. The optimal reaction conditions for recombinant PoGal3 were found to be pH 5.0 and 40°C, consistent with fungi preferring to grow under mildly acid conditions. Previous research has demonstrated many glycosides from fungi exhibiting better activity under acid conditions, such as an α-L-arabinofuranosidase from *Penicillium chrysogenum* [38], β-galactosidase from *Aspergillus* [39] and endo-β-1,6-galactanase from *Trichoderma viride* [40]. β-1,3-Galactanases are also most often active under acidic conditions [25, 41]. As for temperature, most enzymes belonging to GH43 family show optimal activity over the temperature range of 40-50°C [25, 42, 43]. Thermostability, along with the highly efficient expression of PoGal3 in *P. pastoris* suggests that recombinant PoGal3 is likely to be valuable to the pharmaceutical industry.

In this study, PoGal3 is active against β-1,3-galactan. To date, most characterized exo-β-1,3-galactanases have considerable activity toward β-1,3-galactan [18-26]. Moreover, all enzymes identified so far belong to the GH 43 family, like PoGal3 identified here. Furthermore, based on multiple-sequence alignment and phylogenetic analysis, PoGal3 has the greatest similarity to Fo1,3Gal, which has a “GH43-6 domain” and a “CBM35 galactosidase-like” domain [20]. Research has shown that GH43 family members have three conserved acidic residues that function in hydrolysis of substrate, including a general acid, a general base, and a pKa modulator of the general acid [44]. For example, the three conserved amino acid residues in Fo1Gal3, namely Glu103, Glu205, and Asp159, are also conserved in PoGal3 as Glu102, Glu204, and Asp158. In this regard, PoGal3 and Fo1,3Gal have similar functions. Both of them bind β-1,3-galactan and release galactose and galactooligomer, whereas they do not hydrolyze LWAG. This suggests that the side chains attached to β-1,3-galactan backbones prevent the enzyme from accessing the backbone. Therefore, PoGal3 may be suitable for the hydrolysis of arabinogalactans that have no side chains.

In this study, an exo-1,3-galactanase named PoGal3 from *P. oxalicum* sp.68 was cloned and characterized. The gene was successfully expressed in *P. pastoris* GS115, and the optimal temperature and pH of the recombinant PoGal3 were found to be 40°C and 5.0. This is the first GH43 family exo-1,3-galactanase reported from *P. oxalicum*. PoGal3 can effectively hydrolyze β-(1-3)-galactosidic linkages in arabinogalactans, releasing galactose and galactooligomers. This indicates that PoGal3 has the potential to become a new tool in the structural analysis of polysaccharides.

## Supplemental Materials

Supplementary data for this paper are available on-line only at http://jmb.or.kr.

## Figures and Tables

**Table 1:** GC-MS analysis of the methylated products of AG-P-I.

Methylated sugars	Type of linkage	Molar ratio (%)	Mass fragments (m/z)
2,4,6-Me_3_-Gal*p*	1,3-linked Gal*p*	58.5	87, 101, 117, 129, 161, 233, 277
2,3,4-Me_3_-Gal*p*	1,6-linked Gal*p*	9.4	71, 87, 99, 101, 117, 129, 161, 189, 233
2,3,4,6-Me_4_-Gal*p*	t-linked Gal*p*	20.2	71, 87, 101, 117, 129, 145, 161, 205
2,5-Me_2_-Ara*f*	1,3-linked Ara*f*	3.4	87, 99, 117, 129, 159, 201, 233

**Table 2:** Effects of metal ions and chemical agents on the activity of PoGal3.

Metal ions or reagents (50 mM)	Relative activity (%)^a^
NaCl	95.0% ± 0.5%
KCl	102.1% ± 1.2%
MgCl_2_	59.4% ± 0.8%
CuCl_2_	15.8% ± 0.1%
FeCl_3_	80.9% ± 2.5%
BaCl_2_	91.8% ± 1.0%
CaCl_2_	95.7% ± 0.6%
HgCl_2_	5.4% ± 2.4%
MnCl_2_	68.4% ± 1.6%
ZnCl_2_	128.6% ± 4.7%
LiCl	87.6% ± 0.8%
NiCl_2_	64.7% ± 2.4%
EDTA	48.5% ± 0.1%
DTT	106.9% ± 0.1%
SDS	--

^a^The activity assayed in the absence of cations or reagents was taken as 100%.

Results are presented as means ± SD (*n* = 3).

**Table 3:** Substrate specificity of recombinant PoGal3 towards different polysaccharides.

Substrates (5 mg/ml)	Relative activity (%)^a^
AG-P-I	100% ± 0%
β-1,4-Galactan from potato	-
Acacia	-
Arabinogalactan from larch wood (LWAG)	7.4% ± 0.1%
β-1,3-Glucan	25.6% ± 0.1%
Carboxymethyl cellulose (CMC)	-
Laminarin	8.3% ± 0.1%
Oat xylan	-
Wheat arabinoxylan	-
Debranched arabinan (sugar beet)	-
Arabinan (sugar beet)	-

^a^Activity is expressed as percentage of that towards AG-P-I taken as 100%.

## References

[1] Goellner EM, Utermoehlen J, Kramer R, Classen B (2011). Structure of arabinogalactan from *Larix laricina* and its reactivity with antibodies directed against type-II-arabinogalactans. Carbohydr. Polym..

[2] Prescott JH, Groman EV, Gulyas G (1997). New molecular weight forms of arabinogalactan from *Larix occidentalis*. Carbohydr. Res..

[3] Tang S, Jiang M, Huang C, Lai C, Fan Y, Yong Q (2018). Characterization of arabinogalactans from Larix principis-rupprechtii and their effects on NO production by macrophages. Carbohydr. Polym..

[4] Currier NL, Lejtenyi D, Miller SC (2003). Effect over time of in-vivo administration of the polysaccharide arabinogalactan on immune and hemopoietic cell lineages in murine spleen and bone marrow. Phytomedicine.

[5] Beuth J, Ko HL, Schirrmacher V, Uhlenbruck G, Pulverer G (1988). Inhibition of liver tumor cell colonization in two animal tumor models by lectin blocking with D Galactose or arabinogalactan. Clin. Exp. Metastasis.

[6] Enriquez PM, Chu J, Josephson L, Tennant BC (1995). Conjugation of Adenine arabinoside 5'-Monophosphate to arabinogalactan: Synthesis, characterization, and antiviral activity. Bioconjug. Chem..

[7] Riede L, Grube B, Gruenwald J (2013). Larch arabinogalactan effects on reducing incidence of upper respiratory infections. Curr. Med. Res. Opin..

[8] Scigelova M, Singh S, Crout DHG (1999). Glycosidases-a great synthetic tool. J. Mol. Catal. B Enzym..

[9] Müller M, Calvert M, Hottmann I, Kluj RM, Mayer C (2021). The exo-β-N-acetylmuramidase NamZ from *Bacillus subtilis* is the founding member of a family of exo-lytic peptidoglycan hexosaminidases. J. Biol. Chem..

[10] Yang H, Ichinose H, Yoshida M, Nakajima M, Kobayashi H, Kaneko S (2006). Characterization of a thermostable Endo-β-1,4-galactanase from the Hyperthermophile *Thermotoga maritima*. Biosci. Biotechnol. Biochem..

[11] Wang D, Li K, Wang GZ, Li ZY, Qin XM, Du GH (2018). Establishment of fingerprint of *Astragali Radix* polysaccharides based on endo-1,4-β-malactanase hydrolysis and identification of Astragali Radix of different germplasm resources. Zhongguo Zhong Yao Za Zhi..

[12] Bueren AL, Mulder M, Leeuwen SV, Dijkhuizen L (2017). Prebiotic galactooligosaccharides activate mucin and pectic galactan utilization pathways in the human gut symbiont *Bacteroides thetaiotaomicron*. Sci. Rep..

[13] Ichinose H, Kotake T, Tsumuraya Y, Kaneko S (2006). Characterization of an exo-β-1,3-D-galactanase from *Streptomyces avermitilis* NBRC14893 acting on arabinogalactan-proteins. Biosci. Biotechnol. Biochem..

[14] Lemaire A, Garzon CD, Perrin A, Habrylo O, Trezel P, Bassard S (2020). Three novel rhamnogalacturonan I- pectins degrading enzymes from *Aspergillus aculeatinus*: Biochemical characterization and application potential. Carbohydr. Polym..

[15] Torpenholt S, Poulsen J, Muderspach SJ, Maria LD, Leggio LL (2019). Structure of *Aspergillus aculeatus* β-1,4-galactanase in complex with galactobiose. Acta Crystallogr. F Struct. Biol. Commun..

[16] Okemoto K, Uekita T, Tsumuraya Y, Hashimoto Y, Kasama T (2003). Purification and characterization of an endo-β-(1→6)-galactanase from *Trichoderma viride*. Carbohydr. Re..

[17] Tsumuraya Y, Mochizuki N, Hashimoto Y, Kovac P (1990). Purification of an exo-β-(1→3)-D-galactanase of Irpex lacteus (*Polyporus tulipiferae*) and its action on arabinogalactan-proteins. J. Biol. Chem..

[18] Kotake T, Kitazawa K, Takata R, Okabe K, Ichinose H, Kaneko S (2009). Molecular cloning and expression in Pichia pastoris of a *Irpex lacteus* exo-β-(1→3)-galactanase Gene. Biosci. Biotechnol. Biochem..

[19] Fujita K, Sakaguchi T, Sakamoto A, Shimokawa M, Kitahara K (2014). *Bifidobacterium longum* subsp. longum Exo-β-1,3-Galactanase, an Enzyme for the Degradation of Type II Arabinogalactan. Appl. Environ. Microbiol..

[20] Okawa M, Fukamachi K, Tanaka H, Sakamoto T (2013). Identification of an exo-β-1,3-D-galactanase from *Fusarium oxysporum* and the synergistic effect with related enzymes on degradation of type II arabinogalactan. Appl. Microbiol. Biotechnol..

[21] Ichinose H, Yoshida M, Kotake T, Kuno A, Igarashi K, Tsumuraya Y (2005). An exo-beta-1,3-galactanase having a novel beta-1,3-galactan-binding module from *Phanerochaete chrysosporium*. J. Biol. Chem..

[22] Ishida T, Fujimoto Z, Ichinose H, Igarashi K, Kaneko S, Samejima M (2009). Crystallization of selenomethionyl exo-beta-1,3-galactanase from the basidiomycete *Phanerochaete chrysosporium*. Acta Crystallogr. Sect. F Struct. Biol. Cryst. Commun..

[23] Ichinose H, Kuno A, Kotake T, Yoshida M, Sakka K, Hirabayashi J (2006). Characterization of an exo-beta-1,3-galactanase from *Clostridium thermocellum*. Appl. Environ. Microbiol..

[24] Jiang D, Fan J, Wang X, Zhao Y, Huang B, Liu J (2012). Crystal structure of 1,3Gal43A, an exo-β-1,3-galactanase from *Clostridium thermocellum*. J. Struct. Biol..

[25] Ichinose H, Kotake T, Tsumuraya Y (2006). Characterization of an exo-β-1,3-D-galactanase from *Streptomyces avermitilis* NBRC14893 acting on arabinogalactan-proteins. J. Agric. Chem. Soc. Japan.

[26] Ling NX, Lee J, Ellis M, Liao ML, Mau SL, Guest D (2012). An exo-β-(1→3)-D-galactanase from *Streptomyces* sp. provides insights into type II arabinogalactan structure. Carbohydr. Res..

[27] Hu Y, Yan X, Zhang H, Liu J, Luo F, Cui Y (2018). Cloning and expression of a novel α-1,3-arabinofuranosidase from *Penicillium oxalicum* sp. 68. AMB Express.

[28] Li J, Liu G, Chen M, Li Z, Qin Y, Qu Y (2013). Cellodextrin transporters play important roles in cellulose induction in the cellulolytic fungus *Penicillium oxalicum*. Appl. Microbiol. Biotechnol..

[29] Laemmli UK (1970). Cleavage of structural proteins during the assembly of the head of bacteriophage T4. Nature.

[30] Somogyi M (1952). Notes on sugar determination. J. Biol. Chem..

[31] Tsumuraya Y, Hashimoto Y, Yamamoto S, Shibuya N (1984). Structure of l-arabino-d-galactan-containing glycoproteins from radish leaves. Carbohydr. Res..

[32] Zhang X, Li Y, Bi H, Li X, Ni W, Han H (2009). Total fractionation and characterization of the water-soluble polysaccharides isolated from Panax ginseng C.A. Meyer. Carbohydr. Polym..

[33] Wu D, Cui L, Yang G, Ning X, Sun L, Zhou Y (2018). Preparing rhamnogalacturonan II domains from seven plant pectins using *Penicillium oxalicum* degradation and their structural comparison. Carbohydr. Polym..

[34] Shakhmatov EG, Belyy VA, Makarova EN (2018). Structure of acid-extractable polysaccharides of tree greenery of *Picea abies*. Carbohydr. Polym..

[35] Yao Y, Jian Y, Du Z, Wang P, Kan D (2018). Structural elucidation and immune-enhancing activity of an arabinogalactan from flowers of *Carthamus tinctorius* L. Carbohydr. Polym..

[36] Kotake T, Hirata N, Degi Y, Ishiguro M, Kitazawa K, Takata R (2011). Endo-β-1,3-galactanase from winter mushroom *flammulina velutipes*. J. Biol. Chem..

[37] Ali N, Xue Y, Gan L, Liub J, Longb M (2016). Purification, characterization, gene cloning and sequencing of a new β-glucosidase from *aspergillus niger* be-2. Appl. Biochem. Microbiol..

[38] Sakamoto T, Ogura A, Inui M, Tokuda S, Hosokawa S, Ihara H (2011). Identification of a GH62 alpha-L-arabinofuranosidase specific for arabinoxylan produced by *Penicillium chrysogenum*. Appl. Microbiol. Biotechnol..

[39] Dragosits M, Pflügl S, Kurz S, Razzazi-Fazeli E, Wilson I, Rendic D (2014). Recombinant aspergillus β-galactosidases as a robust glycomic and biotechnological tool. Appl. Microbiol. Biotechnol..

[40] Kotake T, Kaneko S, Kubomoto A, Haque MA, Kobayashi H, Tsumuraya Y (2004). Molecular cloning and expression in *Escherichia coli* of a *Trichoderma viride* endo-beta-(1-->6)-galactanase gene. Biochem. J..

[41] Kato H, Takeuchi Y, Tsumuraya Y, Hashimoto Y, Nakano H, Ková P (2003). In vitro biosynthesis of galactans by membrane-bound galactosyltransferase from radish (*Raphanus sativus* L.) seedlings. Planta.

[42] Liu Y, Huang L, Zheng D, Xu Z, Li Y, Shao S (2019). Biochemical characterization of a novel GH43 family β-xylosidase from *Bacillus pumilus*. Food Chem..

[43] Jordan DB, Wagschal K, Grigorescu AA, Braker JD (2013). Highly active β-xylosidases of glycoside hydrolase family 43 operating on natural and artificial substrates. Appl. Microbiol. Biotechnol..

[44] Vandermarliere E, Bourgois TM, Winn MD, Van Campenhout S, Volckaert G, Delcour JA (2009). Structural analysis of a glycoside hydrolase family 43 arabinoxylan arabinofuranohydrolase in complex with xylotetraose reveals a different binding mechanism compared with other members of the same family. Biochem. J..

