# Supplementary material for: Molecular Cloning and Characterization of a Novel Exo-β-1,3-Galactanase from Penicillium oxalicum sp. 68
Source: J Microbiol Biotechnol. 2022 Jul 8;32(8):1064–71. doi: 10.4014/jmb.2204.04012 (PMC9628948; doi:10.4014/jmb.2204.04012)
Supplement: Supplementary file 1 [file jmb-32-8-1064-supple.pdf]

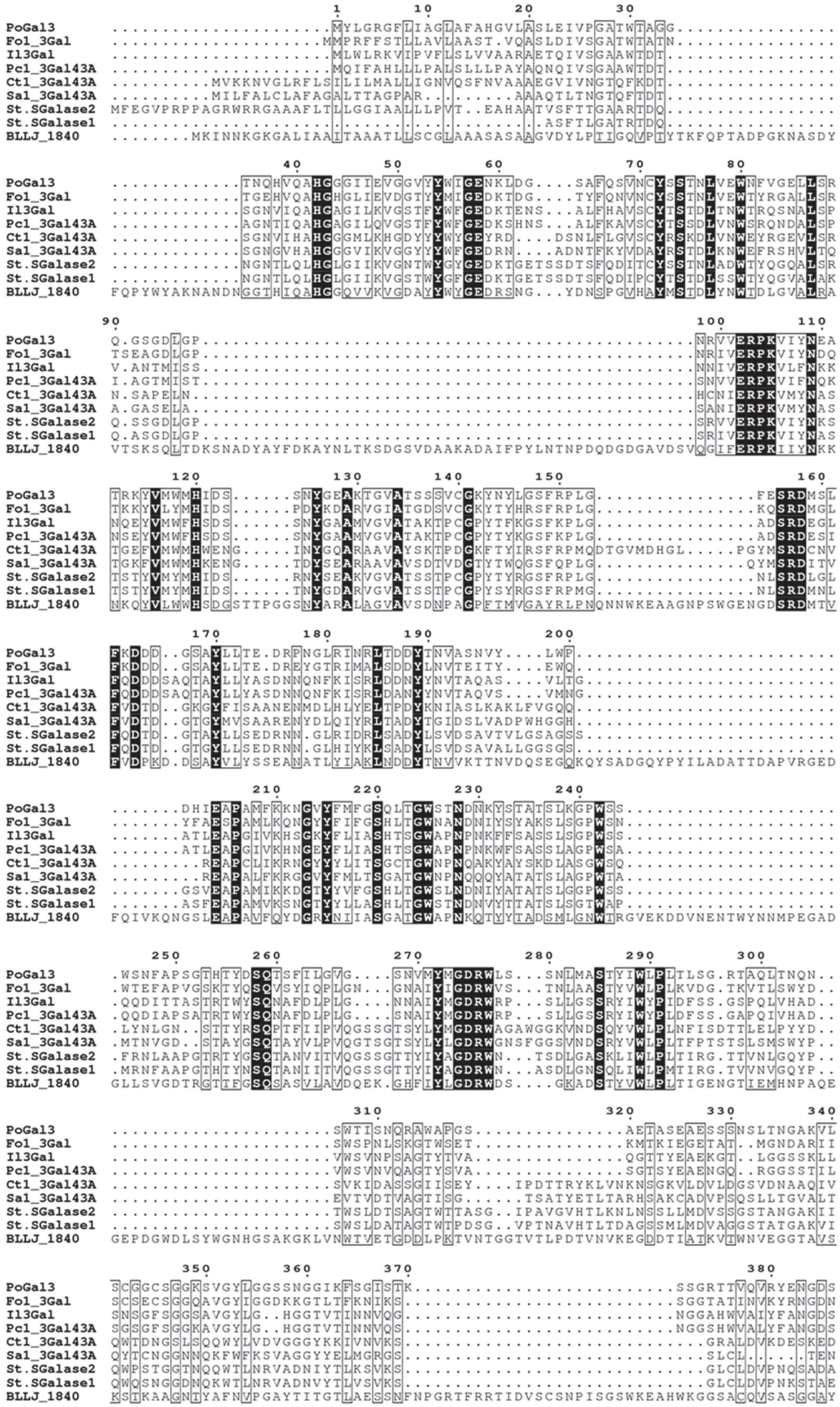
PoGal3
Fo1_3Gal
I13Gal
Pc1_3Gal43A
Ct1_3Gal43A
Sa1_3Gal43A
St.SGalase2
St.SGalase1
BLLJ_1840

```
                  390       400       410                 420       430       440
PoGal3        TQRYATVNVNGKSQILAFVPSKDG........NTPSSST.LNVDLNAGNSNVITISGYQGGWAPDVDRLLVP
Fo1_3Gal      GSRYATVNVNGESQKLAFLSTSHL........SQTGLSR.GFFDLKEGSDNTITIS.NGDGWGPDVDALMPP
Il3Gal        TYRNVTVSVNGGSSVLVDQPDSGG........GGVVISVPVKVNLNNG.ANSITFGSGQSNYAADLDKIIVY
Pc1_3Gal43A   TYRNVTVSVNGGPSVLVDQPDSGG........GNVVISVPVKLNLNSG.ENSITFGSGQSNYAADLDKIIVY
Ct1_3Gal43A   GGVLIQYTSNGGYNQHWKFTDIGD...GYYKISSRHCGKLIDVRKWSTEDGGIIQQWSDAGGTNQHWKLVLV
Sa1_3Gal43A   VTAVTQENCTAATSQQWSLTTSG....GYVSLKSRASGECLDVSGASTANSAALITYTCNGGTNQQWTRGT.
St.SGalase2   GVQLEQWTCNGGTNQQWFLDLVGSYTAGTDMLASVGSDLEIGVANASTTSRAAVEQETGTGATSQQWSLS..
St.SGalase1   NVQLQQWTCNGGANQQFAADLVGSLTGTKFMLVNVNSGLNIGVDSSSTTAGAGVVQLTGTGATSQQWTLS..
BLLJ_1840     DFTITDNANRGVWTDRNEGSAVYQPDALDVNETLETTVKPLDLGGNGDPRAGLVVRNGLADANGGKGYATLL

             450
PoGal3        QQ......................................................................
Fo1_3Gal      AA......................................................................
Il3Gal        ........................................................................
Pc1_3Gal43A   ........................................................................
Ct1_3Gal43A   SSPEPSPSPSPQVVKGDVNGDLKVNSTDFSMLRRYLLKTIDNFPTENGKQAADLNGDGRINSSDLTMLKRYL
Sa1_3Gal43A   ........................................................................
St.SGalase2   ........................................................................
St.SGalase1   ........................................................................
BLLJ_1840     ASLSGVYMQYDSNADGYIDKETSHVGTGFGDQVQLKLERISTDTLKGYWRASANDEWQDVATVTLTGADVTG

PoGal3        ........................................................................
Fo1_3Gal      ........................................................................
Il3Gal        ........................................................................
Pc1_3Gal43A   ........................................................................
Ct1_3Gal43A   LMEVDL..................................................................
Sa1_3Gal43A   ........................................................................
St.SGalase2   ........................................................................
St.SGalase1   ........................................................................
BLLJ_1840     LDAGAFATSNSNAGAFTVAFNGTAFGSQTAAVESIAAKGPETTIAKRQTLAHKDVTVTATLTNGKTRVLEPD

PoGal3        ........................................................................
Fo1_3Gal      ........................................................................
Il3Gal        ........................................................................
Pc1_3Gal43A   ........................................................................
Ct1_3Gal43A   ........................................................................
Sa1_3Gal43A   ........................................................................
St.SGalase2   ........................................................................
St.SGalase1   ........................................................................
BLLJ_1840     EYTLEGFDTTKLGEQTVTVRLVTDSSVTATLTVTVESNLARLFCSSAAASKYEPASSWASASTADLTCDNNL

PoGal3        ........................................................................
Fo1_3Gal      ........................................................................
Il3Gal        ........................................................................
Pc1_3Gal43A   ........................................................................
Ct1_3Gal43A   ........................................................................
Sa1_3Gal43A   ........................................................................
St.SGalase2   ........................................................................
St.SGalase1   ........................................................................
BLLJ_1840     STNWSNWGTGDTSPWLSYTFDKAYQLGKLSVAVDKAKGEAAPKSFTVSYLAEDNATWTDATLPAVTVNGAAG

PoGal3        ........................................................................
Fo1_3Gal      ........................................................................
Il3Gal        ........................................................................
Pc1_3Gal43A   ........................................................................
Ct1_3Gal43A   ........................................................................
Sa1_3Gal43A   ........................................................................
St.SGalase2   ........................................................................
St.SGalase1   ........................................................................
BLLJ_1840     AVTEADVSALPATKGIRLNFTYADGNDYAKIAEVRIAEGEATPEPQPSSNANLADLTVDGKTVDGFSADITE
```

Supplementary Figure 1. Comparison of the amino acid sequences of enzymes from GH43. Shown was an alignment of the deduced amino acid sequences of exo-β-1,3-galactanase Il3Gal (GenBank: BAH29957.1), BLLJ_1840 (GenBank: BAJ67504.1), Fo1,3Gal (GenBank: BAG80558.1), Pc1,3Gal43A (GenBank: BAD98241.1), Ct1,3Gal43A (GenBank: ABN51896.1), Sa1,3Gal43A (GenBank: BAC69820.1) and SGalase2/SGalase1 (GenBank: AFH55135.1 / AFH55134.1).
